# Draft Genome Sequence of a *Streptococcus agalactiae* Strain Isolated from a Preterm Neonate Blood Sepsis Patient at the Royal Infirmary, Edinburgh, Scotland

**DOI:** 10.1128/genomeA.00875-14

**Published:** 2014-09-04

**Authors:** K. A. Kropp, A. Lucid, J. Carroll, V. Belgrudov, P. Walsh, B. Kelly, C. Smith, P. Dickinson, A. O’Driscoll, K. Templeton, P. Ghazal, R. D. Sleator

**Affiliations:** a Department of Biological Sciences, Cork Institute of Technology, Bishopstown, Cork, Ireland; b Nsilico, Cork, Ireland; c Department of Computing, Cork Institute of Technology, Bishopstown, Cork, Ireland; d Division of Pathway Medicine, University of Edinburgh, Edinburgh, United Kingdom; e Microbiological Diagnostic Unit, Royal Infirmary, University of Edinburgh, Edinburgh, United Kingdom

## Abstract

Herein, we report the draft genome sequence of *Streptococcus agalactiae* ED-NGS-1000, cultivated from a blood sample taken from a preterm neonate blood sepsis patient at the Royal Infirmary, Edinburgh, Scotland, United Kingdom.

## GENOME ANNOUNCEMENT

*Streptococcus agalactiae*, a member of the group B streptococci, is a Gram-positive pathogen linked to early-onset sepsis in neonates (1–4). Preterm neonates are a highly susceptible patient group, due to their immature immune status and the invasive procedures they are subjected to in neonatal intensive care unit (ICU) settings (1, 5, 6). Rapid detection of blood sepsis and characterization of the causative agent are critical to enable proper treatment (7–9). In the ClouDx-i project, we aim to extend knowledge of currently circulating pathogens linked with neonatal blood sepsis to inform the development of improved molecular diagnostic assays. Herein, we present the draft genome of a *Streptococcus agalactiae* strain isolated from a preterm neonate. Positivity for blood sepsis and identification to the species level were confirmed by classical microbiological identification and characterization techniques.

Following overnight growth at 37°C on Luria broth (LB) agar, genomic DNA was isolated using Qiagen genomic tips (Venlo, Limburg, Netherlands). Genomic DNA fragments, ranging in size from 2 to 10 kb, were produced by sonication. The resulting fragments were then used to produce a non-size-selected genome library with the Nextera mate pair kit (Illumina, San Diego, CA). Libraries were sequenced on an Illumina MiSeq using a MiSeq reagent kit v. 3. Genomic sequence assembly, analysis, and automated reporting were achieved using Simplicity (10). This approach produced 3,295,403 total reads, resulting in an estimated average 307-fold coverage of the genome. The average G+C content was 35.71%. A *de novo* assembly pipeline based on the Spades 3.10 assembly tool was used with k-mers K21, K33, K55, K77, K99, and K127 nucleotides in length, resulting in a total of 135 contigs, of which 8 were >1,000 bp, representing 97.40% of the total sequence information, with the largest contig being 2,181,383 bp in length. The genome was then initially annotated using Prokka (11) and the identified 16S rRNA genes were used to confirm the species as *Streptococcus agalactiae*. A scaffold of the genome was produced with Contiguator2 and we attempted to identify the closest related strain by BLASTing the scaffold against the NCBI database, returning *S. agalactiae* A909 and 09mas018883 as the closest though not absolutely identical sequences, as evidenced by some insertions and deletions in the genome. The genome was then screened using Glimmer 3 (12), revealing 2,125 open reading frames (ORFs). The predicted ORFs were compared to the Uniprot Trembl database (13) using BLASTp. A total of 1,338 ORFs were mapped to the protein database and putative virulence factors were identified by comparison with the VFDB (14) and Victors databases. We used 75% amino acid sequence identity as a cutoff while considering only alignments longer than 100 amino acids, identifying 38 hits.

Samples were handled in accordance with local ethical approval by the ethics committees of the NHS Lothian SAHSC Bioresource and NHS R&D office (project identification [ID] 2011/R/NE/01) and the HSS BioResource (RequestID 13/ES/0126).

### Nucleotide sequence accession numbers.

This whole-genome shotgun project has been deposited at DDBJ/EMBL/GenBank under the accession number JPOV00000000. The version described in this paper is version JPOV01000000.
